# Peripheral Nerve Stimulation for Lower Extremity Pain

**DOI:** 10.3390/biomedicines10071666

**Published:** 2022-07-11

**Authors:** Clayton Busch, Olivia Smith, Tristan Weaver, Jayesh Vallabh, Alaa Abd-Elsayed

**Affiliations:** 1 Department of Anesthesiology, The Ohio State University Wexner Medical Center, Columbus, OH 43214, USA; clayton.busch@osumc.edu (C.B.); tristan.weaver@osumc.edu (T.W.); jayesh.vallabh@osumc.edu (J.V.); 2 Wright State University Boonshoft School of Medicine, Dayton, OH 45324, USA; smith.2651@wright.edu; 3 Department of Anesthesiology, University of Wisconsin School of Medicine and Public Health, Madison, WI 53792, USA

**Keywords:** neuromodulation, neurostimulation, peripheral nerve stimulation, lower extremity pain, neuropathy

## Abstract

Peripheral nerve stimulation (PNS) is rapidly increasing in use. This interventional pain treatment modality involves modulating peripheral nerves for a variety of chronic pain conditions. This review evaluated its use specifically in the context of chronic lower extremity pain. Studies continue to elucidate the utility of PNS and better define indications, contraindications, as well as short- and long-term benefits of the procedure for the lower extremity. While large, prospective evidence is still lacking, the best available evidence suggests that improvements may be seen in pain scores, functionality, and opioid consumption. Overall, evidence synthesis suggests that PNS for the lower extremities may be a viable option for patients with chronic lower extremity pain.

## 1. Background

**Figure 1:**
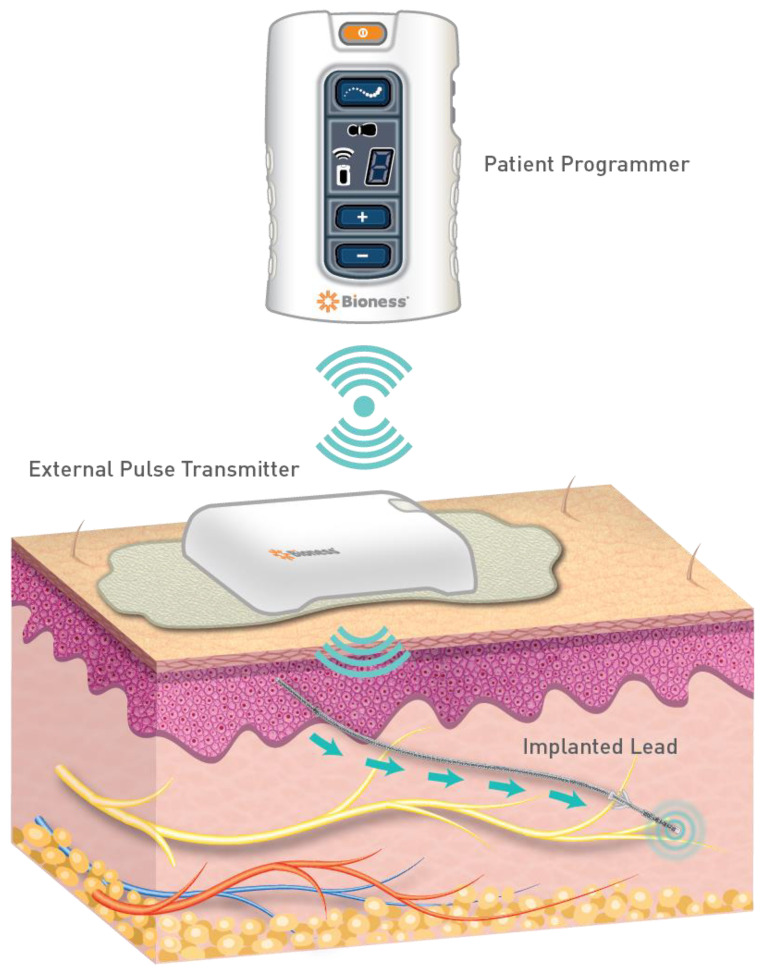
PNS is a system that applies electrical nerve stimulation by placing leads in close proximity to a named nerve. A pulse generator produces stimulation via the lead with the settings controlled by a patient via a programmer. The pictured example exhibits a Bioness Stimrouter PNS system (image courtesy of Bioventus).

Peripheral nerve stimulation (PNS) is on the rise as an interventional pain treatment modality as evidence continues to mount regarding its therapeutic potential [1,2,3,4]. Interestingly, PNS actually predates spinal cord stimulation (SCS) as the first application of the gate theory of pain when Wall and Sweet applied stimulation to their own trigeminal nerve branches in the 1960s [5]. SCS later emerged as the mainstream application, however, new devices are now specifically tailored to target the peripheral nerves [1,3,6]. PNS involves the subcutaneous placement of electrode leads in close proximity to nerves that are in the distribution of the patient’s pain using fluoroscopic or ultrasound guidance, Figure 1. The precise indications for the procedure are still growing but evidence has revealed promise in treating chronic headaches, post-amputation pain, chronic pelvic pain, and chronic low back pain [2]. More recently, PNS has been used to treat lower extremity pain, however, evidence-based guidance regarding its use for this has yet to be synthesized. Thus, the aim of this evidence-based review was to provide physicians with key data for the use of PNS in patients with lower extremity pain.

## 2. Etiology and Epidemiology of Lower Extremity Pain

For the purposes of this paper, the lower extremity is defined as the anatomy inferior to the iliac crests excluding the pelvis and perineal structures as well as the low back. Neuropathic pain is particularly common in the world of chronic pain, and this is no different for the lower extremity. Neuropathic pain is defined as “an unpleasant sensory and emotional experience associated with, or resembling that associated with, actual or potential tissue damage” by the International Association for the Study of Pain (IASP) [7]. The etiologies of lower extremity pain targeted by PNS may result from nerve damage associated with trauma, iatrogenic injury, nerve compression, and amputation (as seen in neuropathic pain); it may also be secondary nociceptive processes associated with tissue damage such as acute post-operative pain [8,9,10,11,12,13,14]. Exciting preclinical work continues to uncover the basic sciences of neuropathic pain [15,16,17]. The focus of this clinical review, however, will emphasize the application of an emerging technology specifically in the context of lower extremity pain.

According to the CDC, lower extremity pain is the second most common cause of pain, affecting nearly one-third of all patients [18]. Unfortunately, when left untreated, patients with lower extremity pain are at risk for musculoskeletal impairment, diminished quality of life, and increasing health care costs. Notably, these findings disproportionately affect individuals coming from lower socioeconomic backgrounds and adults older than 65 [18]. Thus, the development of effective treatment modalities for lower extremity pain is also a matter of healthcare equality.

## 3. Proposed Mechanisms of Analgesia

The exact mechanism of PNS has yet to be fully explained, but there are multiple postulated mechanisms. The foundational work by Melzack and Wall’s “gate theory of pain” is critical to understanding neuromodulation techniques and devices; this is also true for PNS. A blend of both central and peripheral mechanisms appear to be involved with PNS therapy, which stems from Melzack and Wall’s “gate theory of pain.” [19,20]. Central mechanisms were suggested by EEG evidence in an experiment where volunteers were exposed to a laser that selectively excited Aδ fibers and C fibers in the distribution of the left radial nerve. The study was composed of three groups: PNS of the ipsilateral (left) radial nerve, PNS of the contralateral (right) radial nerve, and a control group that was not treated with PNS. Outcomes were measured using the latency of the N2 signal and amplitude of laser evoked potentials (LEPs) on EEG [21]. LEPs are specific EEG characteristics used to measure pain response. The N2 signal is part of an LEP where an increase in amplitude is consistent with a worse response to a painful stimulus while latency is consistent with a more mild pain experience [22,23]. In this study, ipsilateral PNS resulted in increased latency of N2 signals while contralateral PNS and the control group failed to show an effect on signal latency. Notably, both ipsilateral and contralateral radial nerve stimulation resulted in the decreased amplitude of LEPs suggesting central mechanisms of analgesia were at work [21]. EEG captured the presence of an effect, but the precise mechanisms of central analgesia remain to be described. Notably, positron emission tomography showed increases in blood flow to the contralateral somatosensory cortex in PNS but not SCS, suggesting differences in the mechanisms of analgesia between these modalities [24].

Animal models have evidenced mechanisms of analgesia at the level of the spinal cord during low-frequency stimulation resulting in long-term depression of excitatory postsynaptic potentials in the susbtantia gelatinosa. A reduction in C-fiber activity and improvements in endogenous pain attenuation through inhibition of dorsal wide dynamic range neurons at the spinal cord and dorsal root were also noted. These suggest effects via the spinal cord level even beyond the action of inhibitory interneurons at the dorsal root ganglion [25,26]. In contrast, peripherally, Aδ fiber “fatigue” with PNS and “excitation failure of A and C fibers” with repeated stimulation appears to occur [26,27]. Other peripheral mechanisms could also include reduced ectopic discharges, downregulation of neurotransmitters, endorphins, and local inflammatory mediators [27]. Nerve growth and regeneration could also be promoted with electrical stimulation [2]. Neuromodulation modalities may act through a variety of mechanisms occurring at different levels, but they share a converging pathway through neuroplasticity [4].

## 4. Therapeutic Role of PNS and SCS

Treatment of lower extremity neuropathy begins with conservative measures which can include massage, rest, topical lidocaine, and pharmacological measures such as tricyclic antidepressants, selective serotonin–norepinephrine reuptake inhibitors (duloxetine, venlafaxine), calcium channel α2-δ ligands (gabapentin, pregabalin), and then opiates after prior pharmacologic measures remain ineffective [28,29]. If conservative treatment fails to provide relief, then PNS is often considered the next therapeutic option. Table 1 provides a general outline of treatment for chronic neuropathic pain. A more comprehensive description of pharmacologic therapies is described by Baron and colleagues [28]. Specifically, the Neuromodulation Appropriateness Consensus Committee (NACC) recommends neuromodulation in patients who have failed to have acceptable relief “with reasonable efforts and/or who have unmanageable side effects with their current conservative treatment regimen” [1]. For patients who are deemed adequate candidates for PNS, a trial of therapy should be performed prior to device implantation. Patients with pain improvements in the trial phase that is 50% or greater are considered to have had a successful trial. Improvement in activities of daily living or quality of life may also be considered as an alternative definition of success, as determined by a rehabilitation specialist [1]. A patient meeting these criteria, and lacking any contraindications, should have a discussion with their physician detailing the risks, benefits, and alternatives prior to moving forward with a PNS procedure. Indeed, PNS for lower extremity neuropathy appears to reduce patient opioid utilization [30]. Broadly, PNS for lower extremity pain has already been validated by a prospective, multicenter, randomized, double-blinded, partial crossover study in which PNS was found to be effective for lower extremity pain (level II evidence, per Sackett’s description of levels of evidence) [6,31]. Sackett describes five levels of evidence (grade I–grade V). This is further broken down in to grades of evidence. Level I evidence consists of large, randomized trials with clear cut results. Level II has small, randomized trials with uncertain results. Level III has nonrandomized, contemporaneous controls. Level IV has no controls or historical controls while level V evidence has no controls (such as case series). Level I evidence fits the category of grade A evidence. Level II is grade B evidence, and levels III–V are considered grade C [31]. The content of this review evaluated the evidence for the individual, named nerves of the lower extremity.

The focus of the current paper is to delineate the clinical uses of PNS. Given that SCS has some application in lower extremity pain it will also be described, but more briefly. SCS involves placing leads near the spinal cord within the epidural space. Electrical stimulation is applied to the corresponding nerve levels that incite the patient’s pain. Indications for SCS include failed back surgery syndrome (FBSS) or postlaminectomy pain, complex regional pain syndrome (CRPS), painful diabetic neuropathy, and chronic intractable pain of the lower back and lower limb; meanwhile, PNS is typically reserved for an identifiable lesion in a peripheral nerve [32]. Of note, Oswald and colleagues found PNS to be effective for patients where SCS was ineffective [8]. SCS followed a trajectory reminiscent of what is being observed in PNS today as “indications for its use have grown as this therapeutic modality has become better understood and mechanisms of its delivery have evolved and improved” [32].

Conventional SCS strategies worked better for the radiating component of back pain that went into the limbs but was less effective for axial low back pain. Conventional SCS stimulation occurs in a biphasic pattern within the limits of perception (usually around 100 Hz) that induces paresthesia over the region of pain [1,32]. Innovation in novel stimulation strategies has led to new options such as burst stimulation and high-frequency stimulation [1,32]. Burst stimulation delivers 40 Hz bursts with five spikes delivered at 500 Hz within these bursts. The burst stimulation strategy causes less paresthesias and appears to work better for some patients than conventional stimulation [1]. High-frequency stimulation uses stimulation frequencies on the scale of kilohertz (KHz). High-frequency stimulation is unique in that it does not generate paresthesia because the stimulus is above the threshold of sensation. It also treats axial back pain, which was not well treated using conventional stimulation methods [1,32]. Novel stimulation techniques not only increased the applicability of SCS by broadening its indications but also reduced treatment failure by providing a paresthesia-free option to patients that would otherwise be unable to tolerate this side effect [32].

Nearly all the studies for PNS describe stimulation methods similar to conventional SCS stimulation. Rauck and colleagues describe inducing a “comfortable paresthesia” in the region of pain with 50–100 Hz [33]. Similar settings are found in each of the studies listed in Table 2. Less work is published in terms of high-frequency nerve stimulation for PNS; however, there are inklings that high-frequency stimulation will find a niche in the field of PNS [34,35,36].

## 5. Contraindications and Complications of PNS

Warner’s retrospective case series noted that 10% of cases had infectious complications, most of which resulted in device removal [30]. SCS infection rate is only 2.45% which may suggest the superficial nature of PNS contributed to a higher infection rate [30,37]. Infections were noted a median of 50 days after the procedure. No effect of prophylactic antibiotics was demonstrated. Staph aureus was the most common causative agent. Most infections were unsuccessfully treated with antibiotics and surgical revision. A total of 25% of all PNS devices were removed in this case series. Although, 20% of removed devices were removed due to “complete eradication of pain symptoms” [30]. Notably, infection rates may be significantly lower or higher depending on the stimulator’s design [38]. Other complications noted during a review of the literature include device migration and contact dermatitis [39]. Other reports discuss complications with lead fracture, lead migration, and muscle cramping [40]. Patients may also experience inflammation and pain at the insertion site [41]. Large-scale studies are needed to better profile the risks and complications of these procedures in the lower extremity.

Contraindications for PNS include avoiding patients that are not surgical candidates. If a patient’s comorbidities would make it unsafe to undergo an operation or anesthesia the procedure should not be performed. Local or systemic infection, poorly controlled diabetes, immunosuppression, and anticoagulant therapies that cannot be suspended temporarily for implantation should not undergo the procedure. A failed trial of PNS should not pursue PNS implantation. Additionally, patients not passing a psychological evaluation due to poorly controlled psychiatric co-morbidities should not undergo the procedure [1]. There is an important interplay between pain and mental health where both can affect the other in important ways [28]. A patient with poorly controlled mental health co-morbidities is unlikely to benefit from a PNS procedure.

## 6. Evidence Review for Lower Extremity PNS

### 6.1. Ilioinguinal Nerve

The ilioinguinal nerve originates from the spinal nerve of L1. It supplies sensory input to the inguinal region of the lower extremity [42]. Ilioinguinal neuropathy classically occurs as sequelae after lower abdominal surgery (inguinal hernia repair, appendectomy, or hysterectomy). Treatment starts with conservative management. If conservative management fails, then patients may elect for selective nerve blocks, radiofrequency therapies, or surgical excision. In a case series of three patients who underwent PNS therapy for ilioinguinal neuralgia refractory to pharmacological and surgical interventions, pain levels were reduced by greater than 50%. All patients also reported decreases in pain medicine requirements with PNS and two were able to resume working [43]. Similar results were found in a case by Oswald and colleagues who utilized a Bioness Stimrouter device, Figure 1 [8]. Evidence for PNS in the ilioinguinal nerve is summarized in Table 2.

### 6.2. Genitofemoral Nerve

The genitofemoral nerve originates from spinal nerves L1–L2 [42]. It provides sensory input to the groin and inner thigh. Discomfort with this neuropathy was described in terms of paresthesia, burning pain, and hypoalgesia [44]. The pathology of the condition may be secondary to surgical sequelae, trauma, vasculitis, or infectious processes [9]. Genitofemoral pain treated with PNS has level V evidence as detailed in Table 2. Reported evidence demonstrated a 70–90% reduction in pain. Additionally, patients have reported improved functional ability and decreased opioid usage [8,9,45].

### 6.3. Lateral Femoral Cutaneous Nerve

**Figure 2:**
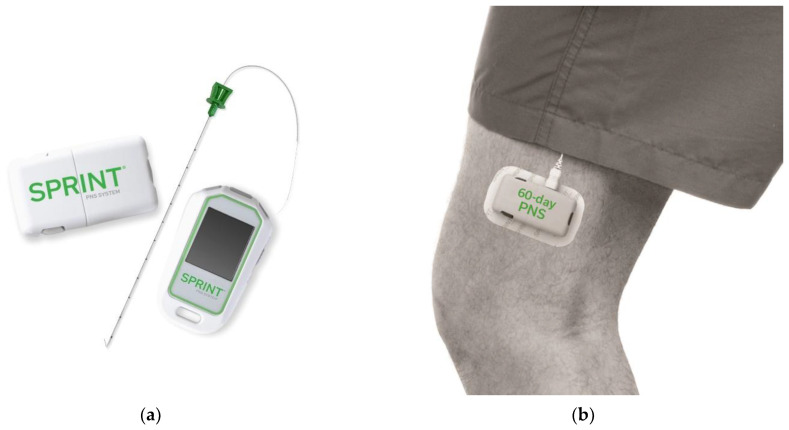
The SPRINT PNS System utilized by Langford and Mauck to treat meralgia paresthetica pain is pictured on the left (**a**). A second picture (**b**) depicts the external implantable pulse generator as it would be worn by a patient (images courtesy of SPR Therapeutics).

The lateral femoral cutaneous originates from spinal nerves L2–L3 and provides sensation to the anterior lateral thigh [46]. Mononeuropathy of the lateral femoral cutaneous nerve is known as meralgia paresthetica. The superficial course of the nerve exposes it to multiple neuropathic etiologies such as obesity, use of tight clothing or belts, and iatrogenic causes such as pelvic surgery [10]. The pain was described as a numb or burning sensation down the lateral thigh that worsens with prolonged standing [10]. For those who fail conservative measures such as weight loss, neuropathic pharmacotherapy, and steroid injections, PNS is used as an effective therapy. The strength of evidence is summarized in Table 2, but for now, evidence is limited to case studies. Specific examples include a case described by Dalal and colleagues where a meralgia paresthetica patient failed multiple therapies prior to PNS including narcotics and steroid injections [47]. A SPRINT PNS lead was placed resulting in an 80% improvement of symptoms at 60 days [47]. In another case report, Langford and Mauck describe SPRINT PNS as effectively treating meralgia paresthetica in a different patient, Figure 2. Specifically, there was a complete resolution of pain symptoms with improved sleep, decreased somnolence, and improved functional ability. The device was explanted at 60 days. Re-evaluation one year after device explanation still demonstrated complete resolution of symptoms [39]. Similar results were achieved with the Bioness Stimrouter system [8].

### 6.4. Femoral and Sciatic Nerves

The femoral nerve originates from spinal nerves L2–L4. The anterior divisions supply sensation to the anteromedial thigh through the anterior cutaneous branches. The posterior divisions of the femoral nerve provide sensation to the medial lower legs and feet through the saphenous nerve and infrapatellar branches of the knee. The sciatic nerve originates from spinal nerves L4–S3. The tibial and peroneal nerves provide sensation to the lower legs and feet [50]. Thus far, much of the literature assessing the effectiveness of PNS for pain in the distribution of the femoral and sciatic nerves was performed in the context of phantom limb pain (PLP). Current treatment for PLP is similar to other neuropathic disorders [11]; however, there has been recent exploration with percutaneous PNS. In a case series by Ruack and colleagues, PLP patients treated with PNS implanted on the femoral and sciatic nerves improved pain scores by 81%. They also had a reduced Pain Disability Index and reported improved quality of life [33]. Another case study observed veterans with PLP who underwent PNS of the sciatic and femoral nerves. The intervention yielded a 50% reduction in pain symptoms. Their relief was again reported at long-term follow-up [51]. Finally, in a larger randomized, double-blinded, placebo-controlled trial, Gilmore and colleagues studied 28 participants with PLP. One group received femoral and sciatic nerve PNS devices and the other received placebo PNS. Therapy for each group was provided for 4 weeks. It was found that patients who received PNS had a significantly greater reduction in pain when compared to the placebo group. Additionally, there was a 71% reduction in the use of opioids while using PNS [40].

PNS of the femoral and sciatic nerves were also studied for use in acute post-operative pain. Specifically, Ilfeld and colleagues studied pain in the foot and knee while identifying an FDA-approved device for this indication [12]. A randomized, double-masked proof of concept trial compared patients undergoing hallux valgus osteotomy surgery that were randomized into two groups: sciatic nerve PNS or sham therapy. After five minutes of stimulation, the PNS group had significantly better analgesia than the sham therapy cohort. PNS was also associated with reduced opioid utilization [41]. Femoral nerve PNS may also yield results in total knee arthroplasty (TKA) and anterior cruciate ligament (ACL) reconstruction [13,42]. Table 2 summarizes the findings for PNS in these nerves for both PLP and acute post-operative pain.

### 6.5. Obturator Nerve

The obturator nerve originates from spinal nerves L2–L4. It functions primarily as a motor nerve, but it does provide a small field of sensory innervation to the medial thigh [50]. One case report (highlighted in Table 2), was identified with a young female suffering from chronic pelvic pain with pubic symphysis dysfunction. She had failed multiple prior treatments for her pain including failed ilioinguinal and iliohypogastric nerve blocks. An obturator nerve block was eventually successful in reducing her symptoms, and she was trialed as a candidate for PNS therapy. Prior to PNS, her pain was poorly controlled on a multimodal pharmacologic regimen inclusive of opioids. PNS leads were then placed laparoscopically. Six months post-implantation the patient was weaned off chronic opioids and at 23 months she described herself as pain-free no longer taking any analgesic medicines. A marked improvement in pain and activity was reported, and the patient was also able to stop taking her antidepressant medication [43].

### 6.6. Saphenous, Infrapatellar Saphenous, and Genicular Nerves

The saphenous nerve, a branch from the femoral, is one of the largest cutaneous branches [52]. The saphenous nerve courses along the medial leg with numerous terminal branches providing sensory input to the medial leg and the knee. Branches to the knee include the infrapatellar saphenous (IPS) nerve, the prepatellar nerve, and the infrapatellar genicular nerve. Pathology to any of these nerves can result in anterior knee pain. Literature on PNS for these nerves is limited to case studies. In one case study, a 58-year-old male with a history of chronic pain related to osteoarthritis received saphenous, IPS, and superior lateral genicular nerve received PNS to achieve pain relief in the lateral knee and reported almost 100% pain relief and improved function after PNS [44]. In another, a 73-year-old female with chronic knee pain refractory to medical management was assessed. The saphenous and superior lateral genicular nerves were targeted with PNS and improvements in pain scores and function were seen three days after the procedure. A 90% improvement in pain was reported after 2 months [45]. A case series reviewing PNS in three saphenous nerve patients reported a 5-point decrease in visual analog scale (VAS) reporting [8]. The findings are summarized in Table 2.

### 6.7. Peroneal Nerve

The common peroneal nerve is a branch of the sciatic nerve and provides sensation to the anterior and lateral parts of the leg and foot [50]. The nerve then branches to the superficial peroneal (SPN), which can be injured after ankle fractures and surgeries due to its superficial nature [53]. This superficial nature also makes it a desirable target for PNS therapy. Again, evidence is limited to case studies. In one case report, a patient with right lateral leg and dorsal foot pain underwent PNS, and two weeks after implantation their pain improved by >80% [46]. In another report, two patients were described that underwent peroneal nerve PNS. During the 3-to-7-day trial, one patient experienced 60% and the other experienced 70% pain relief. Both patients underwent permanent implantation of the stimulator and had sustained relief of symptoms one month and beyond [47]. Oswald and colleagues reported peroneal neuropathy improvements from VAS of 9.0 to 2.3 with an associated 75% improvement in activity [8]. Table 2 represents key highlights on PNS for the peroneal nerve.

### 6.8. Posterior Tibial Nerve

Posterior tibial neuralgia, also known as tarsal tunnel syndrome, is a compression neuropathy that can result in significant foot pain when the tibial nerve and its branches are compressed by the flexor retinaculum [53]. Two studies describe improvement in this condition with PNS. First, in an open-label study, PNS significantly reduced pain in volunteers. Most patients reported a 50% improvement after the first of six sessions and a 99.2% reduction after the last sessions [48]. Another case report described a patient suffering from both posterior tibial nerve neuropathy and sural neuropathy reviewed in greater detail in the sural nerve discussion [49]. The above findings are summarized in Table 2.

Stimulation of the posterior tibial nerve is indicated in an overactive bladder [54]. While not the primary focus of this paper, stimulation of the posterior tibial nerve for overactive bladder is briefly described here for completeness’ sake. The tibial nerve is a branch of the sciatic nerve originating from L4–S3. This nerve is targeted in overactive bladder syndrome as a third-line treatment option [54,55]. Percutaneous stimulation of the posterior tibial nerve has shown efficacy when used alone to treat an overactive bladder. There is further improvement still when combined with anti-muscarinic therapy.

### 6.9. Sural Nerve

The sural nerve is formed by the branches of the tibial and common peroneal nerves [50]. The sural nerve provides sensation to the lateral posterior corner of the leg, lateral foot, and fifth toe. Sural neuralgias, similar to posterior neuralgia, have limited data on PNS. In one case report a 60-year-old man with a history of sural and posterior tibial neuropathy secondary to a motorcycle accident presented with significant pain with mild relief on high-dose opiate therapy. The patient reported a 75% improvement in pain at three months and a 50% improvement at 6 months. He also enjoyed improved mobility [49]. These findings were similar to the 75% pain improvement noted by Oswald when SCS was utilized peripherally for sural neuralgia [8]. Table 2 summarizes these data for PNS in sural nerve pain. Of note, there is currently no gold-standard treatment for sural neuralgia. Treatment should start, as always, with conservative management including massage, rest, and pharmacologic interventions. When these conservative measures fail, PNS may be a suitable option for patients.

## 7. Conclusions

Based on the best available evidence thus far, the quality of evidence for PNS of an individual nerve of the lower extremity appears to be highest for femoral and sciatic nerve stimulation. Evidence for other individual lower extremity nerves is mostly limited to case reports which is the greatest limitation of this paper. While the technology is certainly promising and potentially underutilized [56], further research will better elucidate the short-term and long-term effectiveness of PNS. Of the data available thus far, findings suggest PNS may be an important non-pharmacologic potentiator of analgesia that reduces opioid utilization, improves pain scores, improves functionality, and decreases opioid utilization.

The future of PNS for lower extremity pain is undergoing an exciting period of growth. Device innovations are driving the boom behind PNS. While PNS predates SCS, SCS enjoyed wider implementation, and consequently, greater observation, research, and device innovation [1]. A similar wave of new information is now accruing for PNS. Implantation techniques evolved from invasive neurosurgical operations to outpatient procedures [3]. Earlier PNS procedures implemented SCS leads that were convergently evolved to target the spinal cord, but instead were used off-label to target peripheral nerves, and now there are multiple devices specifically designed for the periphery [1,3,57]. A key innovation in SCS came from the discovery that different types of waveform stimulation were better suited to different types of pain [32]. Other areas of growth could include new targets such as nerve ganglia, targeting specific nerve fascicles to avoid unwanted motor stimulation, and incorporating PNS as an additional arm in multimodal pain control [33,58,59]. Future innovations and applications of PNS for lower extremity pain will be closely linked to its research, especially large randomized controlled trials that are currently lacking in the field.

## Figures and Tables

**Table 1:** A brief overview of treatment modalities for chronic pain.

Treatment Modality	Means of Administration	Duration of Trial
Non-pharmacological therapies	Rest, heat, cold, massage, physical therapy	Per provider and patient discretion
Tricyclic antidepressants	Oral medication	6–8 weeks
SSNRI	Oral medication	4 weeks
Calcium channel α2-δ ligands	Oral medication	4 weeks
Opioid agonists	Oral, transcutaneous, intravenous, or intrathecal administration	4–6 weeks
Spinal cord stimulation	Procedurally placed epidural leads	No consensus, approximately 5-day trial is typical prior to permanent implantation
Peripheral nerve stimulation	Procedurally placed leads in proximity to peripheral nerve	Trial of therapy is not precisely defined. SPRINT PNS System uses a 60-day implementation period prior to removal.

**Table 2:** Individual nerves of the lower extremity described by level of evidence as well as a summary of findings.

Nerve	Evidence Level	Summary of Evidence
Ilioinguinal nerve	Level V	− Four patients decreased pain scores by 5–9 points, decreased pain medicine use, and improved functional ability [8,37].
Genitofemoral nerve	Level V	− Four patients reported 70–90% pain improvement, decreased opioid use, and improved functional ability [8,9,38].
Lateral femoral cutaneous nerve	Level V	− Total of 80–100% improvement in symptoms [8,39].
Femoral and sciatic nerves	Level II	− In an RCT PNS in PLP provided significantly improved benefit over placebo and reduced opioid use by 71% [40]. − Sciatic and femoral nerve PNS may provide relief for acute post-operative pain [12,13,41,42].
Obturator nerve	Level V	− One case report with robust response. Prior to PNS the patient consumed 255mg of morphine daily but was able to discontinue analgesics after PNS [43].
Saphenous, infrapatellar saphenous, and genicular nerves	Level V	− Total of 90–100% improvement in knee pain in 2 case reports [8,44,45]. − Decrease in VAS from 7.7 to 2.7 in another case report [8].
Peroneal nerve	Level V	− Total of 60–80% pain relief or more with PNS [8,46,47]. − 75% improvement in activity [8].
Posterior Tibial nerve	Level V	− Most patients report at least 50% improvement in pain after 6 sessions of PNS [48].
Sural nerve	Level V	− Total of 50–75% improvement in pain at 6 months [8,49]. − 60% improvement in activity [8].

## Data Availability

Not applicable.
